# A sensitive and specific blocking ELISA for the detection of rabbit calicivirus RCV-A1 antibodies

**DOI:** 10.1186/1743-422X-9-182

**Published:** 2012-09-03

**Authors:** June Liu, Peter J Kerr, Tanja Strive

**Affiliations:** 1 Commonwealth Scientific and Industrial Research Organisation, Ecosystem Sciences Division, GPO Box 1700, Canberra, ACT, 2601, Australia; 2 Invasive Animals Cooperative Research Centre, University of Canberra, Canberra, Australia

**Keywords:** Blocking ELISA, RCV-A1, RHDV, Epidemiology, Cross-reactive antibody, Specificity, Sensitivity

## Abstract

**Background:**

Antibodies to non-pathogenic rabbit caliciviruses (RCVs) cross-react in serological tests for rabbit hemorrhagic disease virus (RHDV) and vice versa, making epidemiological studies very difficult where both viruses occur. It is important to understand the distribution and interaction of the two viruses because the highly pathogenic RHDV has been used as a biocontrol agent for wild rabbits in Australia and New Zealand for the past 17 years. The presence of the benign RCV Australia 1 (RCV-A1) is considered a key factor for the failure of RHDV mediated rabbit control in cooler, wetter areas of Australia.

**Results:**

A highly sensitive and specific blocking ELISA was developed for the detection of RCV-A1 antibodies. When sera from rabbits with a known infection history for either RCV-A1 or RHDV were tested, this assay showed 100% sensitivity and no cross-reactivity with RHDV sera (100% specificity).

**Conclusions:**

This new ELISA not only allows the detection of RCV-A1 at a population level, but also permits the serological status of individual rabbits to be determined more reliably than previously described methods. This robust and simple to perform assay is therefore the tool of choice for studying RCV-A1 epidemiology in Australian wild rabbit populations.

## Background

Rabbit calicivirus (RCV) and rabbit hemorrhagic disease virus (RHDV) are positive sense single stranded RNA viruses in the genus *Lagovirus* of the family *Caliciviridae*[1-4]. The non-pathogenic RCVs are of great interest because they are believed to induce cross-protection to the closely related but highly pathogenic RHDV that is used as a biocontrol agent for wild rabbits in Australia and New Zealand [5-11]. RHDV caused mortality rates as high as 95% in dry, warm areas of the Australian continent, but failed to be as effective in wetter, cooler areas [6,12]. The known distribution of a benign calicivirus, RCV-A1, isolated from Australian wild rabbits has so far been consistent with areas where RHDV is less effective [13,14]. The partial cross-protection of RCV-A1 against RHDV was confirmed in experimental infections of domestic rabbits [15], highlighting the need to study the interaction between the two viruses in wild rabbit populations. In Europe, the situation is reversed as rabbits are considered an important part of local ecosystems [16], and the attractive potential of using non-pathogenic RCVs as natural vaccines for conservation of wild rabbit populations makes epidemiological studies of such benign caliciviruses of interest.

Caliciviruses have a well conserved capsid morphology [17]. The amino acid identity of the capsid protein VP60 of RCV and RHDV varies between 86.8% and 91.5%, and there is strong serological cross-reactivity between RHDV and RCVs [6,14,18-21]. Wild rabbit populations in Australia known to be infected with RCV-A1 are also regularly exposed to RHDV, meaning that many wild rabbits have antibodies (Abs) to both viruses. Due to the antigenic similarity and the resulting cross-reactive Abs to the two viruses, studying the seasonal dynamics of one virus in the presence of the other has proved very challenging in the past [10,11,22,23].

Enzyme-linked immunosorbent assays (ELISAs) for the detection of RHDV Abs have been used for many years [2,24], and certain patterns of cross-reactivity of RCV-A1 antibodies in the RHDV ELISAs have been used to infer RCV-A1 serology [7]. However, ELISAs for the specific detection of RCV-A1 Abs were only recently developed [14,25]. As expected, in the highly sensitive RCV-A1 isotype ELISAs for the detection of IgG, IgA and IgM, sera raised against RHDV showed varying levels of cross-reactivity, while a competition ELISA (cELISA-2) for RCV-A1 showed 100% specificity and 76% sensitivity. The cELISA-2 is a useful tool to detect the presence of RCV-A1 at a population level but the low sensitivity means that a large number of samples must be tested to confirm the absence of RCV-A1 [25]. It is therefore of limited value for monitoring the serological status of individual rabbits.

The aim of this study was to develop a more sensitive ELISA that is still highly specific for the detection of RCV-A1 Abs.

## Methods

The production of reagents including RCV-VLP (virus-like particles), anti-RCV-A1 chicken polyclonal antibodies (pAb) and mouse monoclonal antibodies (mAb) has been described previously [25]. Sera from domestic New Zealand white rabbits with a known infection history (RCV-1 to RCV-25, RHDV-1 to RHDV-9) [25] were diluted at 1:10, 1:40, 1:160 and 1:640 in PBS-TY buffer [pH 7.4, PBS with 0.05% Triton X-100 (v/v) and 1% yeast extract (w/v)] for testing.

All ELISAs were carried out in high-binding 96-well microtitre ELISA plates (Serial No. 655061, Greiner Bio-One). Reagents were diluted in PBS-TY buffer for incubation. Unless otherwise stated, incubations were carried out for 1 h at 37°C. After each incubation step, plates were washed 3 times with PBST (PBS with 0.05% Triton X-100) by shaking at 150 rpm for 5 min at room temperature for each washing step. All reagents were added in 50 μl volumes, and the specified concentrations are final concentrations.

The blocking ELISA was performed as follows. The plate was coated with chicken pAbs to RCV-A1 (2.0 μg/ml) diluted in carbonate buffer (pH 9.6) at 4°C overnight. RCV-VLPs were added and incubated, followed by addition of serially diluted rabbit sera. After incubation and washing, anti-RCV-A1 mAb 11F12 was added and incubated. After washing, goat anti-mouse IgG-HRP (horseradish peroxidase) (1.0 μg/ml, Abcam, Cambridge) was added and incubated. After washing, 50 μl of phosphate-citrate buffer (pH 5.0) containing O-phenylenediamine dihydrochloride (Sigma-Aldrich) at 0.5 mg/ml and H_2_O_2_ at 0.02% (v/v) was added. After 5 min incubation at room temperature, the reaction was stopped with 50 μl of 2 M H_2_SO_4_, and the optical density (OD) at 492 nm was measured on a Multiskan Ascent plate reader (Thermo Labsystems). The OD of the negative serum was set to 100%, and the percentage of inhibition of tested sera was calculated as (Inhibition) % = [(OD of negative serum – OD of tested serum)/OD of negative serum] × 100% at the respective dilution. The positive cut-off was set at 25% (see below). Titres are the reciprocal of the final dilution causing >25% inhibition.

All procedures involving production of sera in rabbits were conducted according to the Australian code of practice for the care and use of animals for scientific purposes and were approved by the CSIRO Sustainable Ecosystems Animals Ethics Committee (SEAEC no. 07-01, 08-02, 09-10 and CESAEC no.10-13).

## Results and discussion

**Figure 1:**
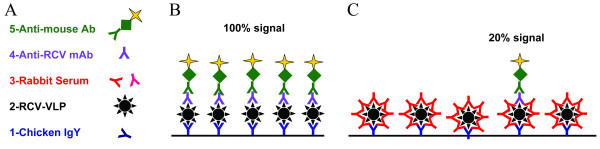
**Design of the blocking ELISA.** (**A**) The five components of the assay are added in succession according to their number. Anti-RCV chicken IgY is used for coating; (**B**) Negative result: negative rabbit serum does not bind to RCV-VLP and shows no inhibition, so the monoclonal antibody gives 100% signal; (**C**) Positive result: RCV specific antibodies in rabbit serum bind to the same epitope as the RCV-A1 specific mAb, resulting in inhibition of the signal.

The setup of this blocking ELISA is shown in Figure 1. Negative rabbit serum will not block the binding of the RCV-A1 specific mAb 11F12 to RCV-VLP, resulting in a positive signal (Figure 1B). When positive RCV-A1 serum is added, the RCV-A1 specific Abs binding to the same epitope as mAb 11F12 will block the binding of mAb 11F12 and the OD is thus reduced (Figure 1C).

**Figure 2:**
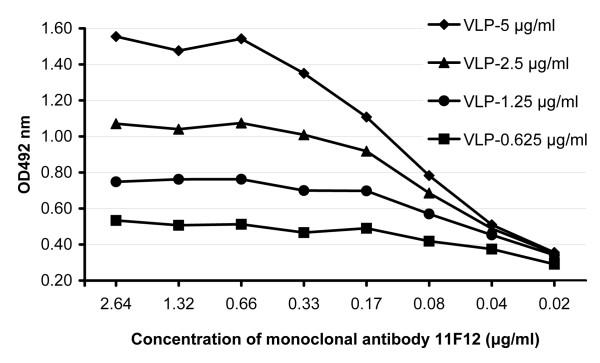
**Optimization of the key reagents for the blocking ELISA.** To determine the optimal concentrations of RCV-VLP and monoclonal antibody 11F12, RCV-VLP and 11F12 were serially diluted and a negative serum was tested at dilution of 1:100.

Among the four reagents used in this blocking ELISA, the concentration of the antigen, RCV-VLP, is the key factor for the sensitivity and specificity of the assay, and the mAb 11F12 and the commercially available anti-mouse IgG-HRP should be used at concentrations that are just sufficient to saturate their respective binding sites. To find the optimal concentrations of RCV-VLP and mAb 11F12 for this assay, serially diluted RCV-VLP and mAb 11F12 were tested by using negative rabbit serum at a dilution of 1:100 (Figure 2). When RCV-VLP was used at 2.5 μg/ml, a plateau occurred when the concentration of mAb 11F12 was higher than 0.33 μg/ml, indicating 0.33 μg/ml of mAb is sufficient to saturate 2.5 μg/ml of RCV-VLP. In the plateau phase, the negative serum gave an OD between 1.0 ~ 1.2. In the final setup of this blocking ELISA, RCV-VLP and mAb 11F12 were used at 2.5 μg/ml (1:1500) and 0.33 μg/ml (1:4000), respectively. The secondary Ab was optimised by testing serial dilutions.

The rationale for choosing this concentration for RCV-VLP is that using more RCV-VLP will reduce the sensitivity of the assay because a higher concentration of RCV-specific Abs in rabbit serum is needed to show inhibition. On the other hand, if less RCV-VLP is used, the negative serum gives a lower OD and some RHDV sera may cause inhibition due to cross-reactive Abs. The mAb 11F12 is specific for RCV-A1 and does not cross-react with RHDV [25] thus inhibition by RHDV sera is most probably due to steric hindrance or conformational changes induced by the binding of cross-reactive Abs to other epitopes adjacent to the epitope specific for mAb 11F12.

To determine the cut-off of this blocking ELISA, sera from domestic rabbits known to be immune to either RCV-A1 (n = 22) or RHDV (n = 9) [25] were tested. When diluted at 1:10, all the RCV-A1 sera showed inhibition higher than 40%, while the inhibitions of the RHDV sera were 10% or lower at the same dilution (Table 1). This assay was repeated three times with the same RCV-A1 and RHDV sera and similar results were obtained. To avoid possible “false-positive” results from RHDV sera, the cut-off of this assay has to be higher than the reduction of OD caused by the cross-reactive Abs in RHDV sera. For this blocking ELISA, a 25% reduction of OD compared with negative serum at the respective dilution was regarded as positive. 

**Table 1:** Comparison of RCV blocking ELISA (bELISA) and competition ELISA (cELISA)

**Sample ID**	**Inhibition at dilution of 1:10 (%)**^a^	**RCV ** **bELISA titre**	**RCV** **cELISA**^b^ **titre**	**RHDV** **cELISA**^b^**titre**
RCV-1^c^	57	160	640	0
RCV-2	56	160	160	0
RCV-3	65	160	40	0
RCV-4	60	320	160	80
RCV-8	42	40	80	0
RCV-9	62	160	320	0
RCV-10	56	160	0	0
RCV-11	72	160	40	0
RCV-12	63	160	20	0
RCV-13	50	160	0	0
RCV-14	51	160	0	0
RCV-15	53	160	0	0
RCV-16	71	160	640	0
RCV-17	59	80	640	0
RCV-18	70	160	80	0
RCV-19	61	160	80	0
RCV-20	43	40	0	0
RCV-21	55	40	0	0
RCV-22	58	40	640	0
RCV-23	66	160	160	0
RCV-24	74	320	160	0
RCV-25	66	160	320	0
RHDV-1	6	0	0	320
RHDV-2	0	0	0	81,960
RHDV-3	0	0	0	20,480
RHDV-4	10	0	0	640
RHDV-5	0	0	0	160
RHDV-6	0	0	0	160
RHDV-7	2	0	0	160
RHDV-8	2	0	0	320
RHDV-9	2	0	0	640
RHDV-W1^d^	7	0	0	80
RHDV-W2	1	0	0	80
RHDV-W3	1	0	0	320
RHDV-W4	1	0	0	80
RHDV-W5	1	0	0	160
RHDV-W6	0	0	0	160
RHDV-W7	6	0	0	80
RHDV-W8	6	0	0	320
RHDV-W9	5	0	0	80
RHDV-W10	8	0	0	40
RHDV-W11	7	0	0	40
RHDV-W12	0	0	0	160
RHDV-W13	0	0	0	640
RHDV-W14	3	0	0	320
RHDV-W15	12	0	0	320
RHDV-W16	6	0	0	320
RHDV-W17	13	0	0	160
RHDV-W18	12	0	0	320
RHDV-W19	23	0	0	640
RHDV-W20	7	0	0	80
RHDV-W21	20	0	0	320
RHDV-W22	14	0	0	640
RHDV-W23	22	0	0	320
RHDV-W24	8	0	0	320
RHDV-W25	12	0	0	320

^a^The inhibition of sera tested at dilution of 1:10 in the blocking ELISA when RCV-VLP was used at 2.5 μg/ml (1:1500) and 0.33 μg/ml (1:4000), respectively. ^b^RCV cELISA and RHDV cELISA were performed according to the RCV and RHDV cELISA-2 in Liu et al. [25]. ^c^Data for RCV cELISA and RHDV cELISA of sera RCV-1 to RHDV-9 are from Liu et al. [25] for comparison. ^d^Sera RHDV-W1 to W25 were sampled from wild rabbit populations proven to be free of RCV-A1 by quantitative PCR and serological tests.

To compare the sensitivity and specificity of this assay to the previously published RCV-A1 cELISA-2 [25], the above sera were tested by endpoint titration. Using the same cut-off (25%), the cELISA-2 failed to detect six of the low level Ab responses to RCV-A1 (underlined in Table 1), while all 22 RCV-A1 sera tested positive in the blocking ELISA, demonstrating the increased sensitivity of this assay. Interestingly, the ELISA titres were not always higher in the blocking ELISA when compared to the cELISA-2. While two- to four-fold changes in ELISA titres between assays are not unusual, the differences in titres for sera RCV-17 and RCV-22 for example cannot be explained by this. Sera RCV-17 and RCV-22 were sampled 27 and 39 days post infection, respectively [25]. It is feasible that some sera from animals with mature immune responses that have a high proportion of high avidity antibodies to the same or overlapping epitopes to the detecting mAb, may have an advantage at higher dilutions in the cELISA-2 setup. The specificity of the blocking ELISA was 100% as all RHDV sera (n = 9) tested negative.

**Figure 3:**
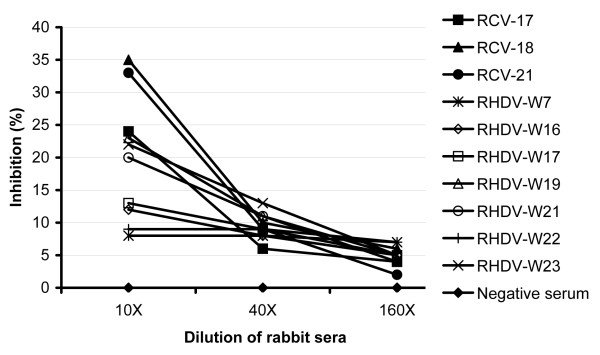
**Titration curves of RCV-A1 and RHDV sera from rabbits in the blocking ELISA.** RCV-17, 18, 21: sera from domestic rabbits infected with RCV-A1. These sera were first diluted with negative serum to produce inhibition levels of 25 – 35%. RHDV-W7 - W23: sera from wild rabbits naturally infected with RHDV showed low levels of cross reactivity. All sera were tested at dilutions of 1:10, 1:40 and 1:160 in PBS-TY.

To confirm the specificity of the assay, sera from wild rabbits naturally infected with RHDV (n = 25) were tested at dilutions of 1:10, 1:40 and 1:160. These samples were all positive to RHDV (Table 1), and were sourced from populations that were known to be free from RCV-A1 by analysing a large number of samples (n > 20) with the RCV-A1 cELISA-2 (data not shown). They all tested negative in the blocking ELISA. Three RHDV sera (RHDV-W19, W21 and W23) showed inhibitions between 20% ~ 23% at a 1:10 dilution. To compare the dilution profiles between low titre RCV sera and RHDV sera from wild rabbits, we first diluted three RCV sera (RCV-17, RCV-18 and RCV-21) in negative serum to produce an inhibition of 25 – 35%, and then compared these diluted RCV sera with sera from wild rabbits. When compared to RCV-A1 sera, the inhibition levels of RHDV sera from wild rabbits were only slightly reduced following serial dilution (Figure 3), indicating that the inhibition was likely due to non-specific binding. To avoid doubtful or false positive results, we therefore recommend that three dilutions of rabbit serum should be tested for the routine detection of RCV-A1 Abs.

The blocking ELISA is much more robust and easier to perform than the cELISA-2 [25]. In the cELISA-2, pre-mixing of rabbit serum with the RCV-A1 specific mAb is necessary, or the addition of rabbit serum has to be followed immediately by the addition of mAb, to provide an equal opportunity for Abs in the rabbit serum and the mAb to bind to the antigen. When testing large numbers of samples, serially diluting and pre-mixing of each rabbit serum with mAb is very time-consuming and, adding rabbit serum and mAb into the same well within a short period of time can be technically challenging. In contrast, the blocking ELISA separates these two steps, resulting in an assay that is much more suitable for high throughput analysis.

In summary, this highly sensitive, specific and easy to perform blocking ELISA for RCV-A1 represents a new serological tool for epidemiological studies of RCV-A1. Due to its high sensitivity, it will allow the detection of RCV-A1 antibodies in rabbits at both population and individual levels.

## Competing interests

The authors declare that they have no competing interests.

## Authors’ contributions

JL designed and carried out all experiments and wrote the manuscript. PK and TS contributed to the experimental design and data interpretation, and critically revised the manuscript. All authors have read and approved the final version of the manuscript.
